# Building Scientific Capability and Reducing Biological Threats: The Effect of Three Cooperative Bio-Research Programs in Kazakhstan

**DOI:** 10.3389/fpubh.2021.683192

**Published:** 2021-10-12

**Authors:** Kenneth B. Yeh, Kairat Tabynov, Falgunee K. Parekh, Elina Maltseva, Yuriy Skiba, Zhanna Shapiyeva, Ablay Sansyzbai, Stefan Frey, Sandra Essbauer, Roger Hewson, Allen L. Richards, John Hay

**Affiliations:** ^1^MRIGlobal, Gaithersburg, MD, United States; ^2^International Center for Vaccinology, Kazakh National Agrarian University, Almaty, Kazakhstan; ^3^EpiPointe LLC, Cary, NC, United States; ^4^Almaty Branch of National Center for Biotechnology at Central Reference Laboratory, Almaty, Kazakhstan; ^5^Scientific Practical Center for Sanitary Epidemiological Expertise and Monitoring, Almaty, Kazakhstan; ^6^Bundeswehr Institute of Microbiology, Munich, Germany; ^7^Bundeswehr Research Institute for Protective Technologies and Chemical Biological Radiological Nuclear (CBRN) Protection, Munster, Germany; ^8^Public Health England, Salisbury, United Kingdom; ^9^London School of Hygiene and Tropical Medicine, London, United Kingdom; ^10^Department of Preventive Medicine and Biostatistics, Uniformed Services University of the Health Sciences, Bethesda, MD, United States; ^11^Jacobs School of Medicine and Biomedical Sciences, Buffalo, NY, United States

**Keywords:** biosecurity, Kazakhstan, global health security, one health approach, vector-borne disease, zoonoses

## Abstract

Cooperative research programs aimed at reducing biological threats have increased scientific capabilities and capacities in Kazakhstan. The German Federal Foreign Office's German Biosecurity Programme, the United Kingdom's International Biological Security Programme and the United States Defense Threat Reduction Agency's Biological Threat Reduction Program provide funding for partner countries, like Kazakhstan. The mutual goals of the programs are to reduce biological threats and enhance global health security. Our investigation examined these cooperative research programs, summarizing major impacts they have made, as well as common successes and challenges. By mapping various projects across the three programs, research networks are highlighted which demonstrate best communication practices to share results and reinforce conclusions. Our team performed a survey to collect results from Kazakhstani partner scientists on their experiences that help gain insights into enhancing day-to-day approaches to conducting cooperative scientific research. This analysis will serve as a basis for a capability maturity model as used in industry, and in addition builds synergy for future collaborations that will be essential for quality and sustainment.

“*Life on Earth is at the ever-increasing risk of being wiped out by a disaster, such as sudden global nuclear war, a genetically engineered virus or other dangers we have not yet thought of*.”

Stephen Hawking

## Background

**Figure 1:**
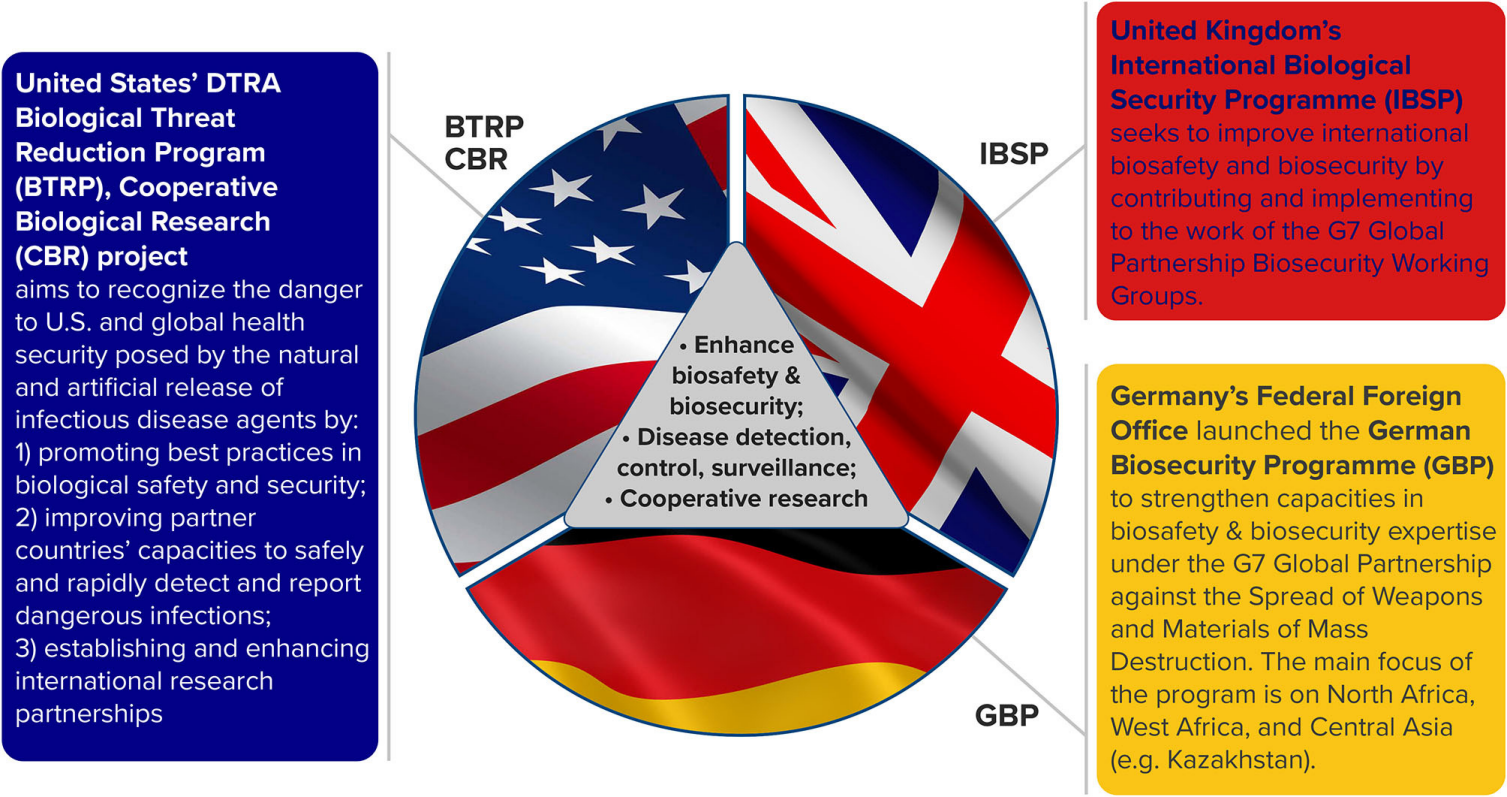
Common aims and objectives for related cooperative research programs in Kazakhstan. These national programs also adhere to international frameworks for biological threat reduction: Biological Weapons Convention (BWC, Article X), World Health Organization International Health Regulations (IHR, 2005), United Nations Security Council Resolution (UNSCR 1540), and G7 Global Partnership Biological Security Deliverables.

Countries from the Former Soviet Union (FSU) including Armenia, Azerbaijan, Georgia, Kazakhstan, Kyrgyzstan, Tajikistan, Ukraine, and Uzbekistan have partnered in various threat reduction, biosecurity, and related programs that engage its scientists in relevant biological research and infectious disease surveillance. The appeal of the Government of Kazakhstan as a partner stems from its work in the former Soviet Union's biological weapons program, the anti-plague surveillance network and a recent history of infectious disease management of hotspots such as those due to anthrax, brucellosis, plague, and tularemia. Earlier, in a compendium, we summarized some aspects of recent cooperative biological research in Kazakhstan, noting its infectious disease surveillance activity, history of scientific achievement, economy, and national research bibliometrics (1). In this paper, we examine the overall impact on scientific capability of three cooperative infectious disease research programs partnering with Kazakhstan (KZ): Germany's Federal Foreign Office's German Biosecurity Programme (GBP), the United Kingdom's (UK) International Biological Security Programme (IBSP), and the United States (US) Defense Threat Reduction Agency's Biological Threat Reduction Program (DTRA BTRP) (Figure 1). Our findings here reflect outputs of enhanced scientific capability and reduced biological threats by Kazakhstan from within and across partner programs.

While each of the programs has specific objectives (Figure 1), the overarching goals are to enhance biosafety and biosecurity, improve disease detection, surveillance and control and engage in cooperative research that will result in sustainable scientific advancements. A major focus of all three cooperative research programs is to build local scientific research capability while reducing biological threats. All three programs have contributed funding to improve infrastructure, cooperative research and related training. DTRA BTRP is a long-standing program with legacy FSU engagements in Armenia, Azerbaijan, Georgia, Kazakhstan, Ukraine, and Uzbekistan; the GBP funds projects in Georgia, Kazakhstan and Ukraine and the IBSP has supported work in Azerbaijan, Georgia, Kazakhstan, Tajikistan, as well as parts of the Middle East and Africa.

Since the early 2000s, the **DTRA BTRP** has funded biological threat reduction in Kazakhstan along three lines of work: biosafety and biosecurity, biosurveillance (i.e., capacities to detect, diagnose, and report disease) and cooperative biological research (CBR). DTRA BTRP has also funded investments for enhancement of facility and infrastructure for BSL-2 Zonal Diagnostic Laboratories (ZDL) in partner countries of the Former Soviet Union (FSU), including Kazakhstan. To reinforce these activities, BTRP recently spent $102 M on the construction of a Central Reference Laboratory (CRL) in Almaty, Kazakhstan. The CRL validation, which includes a biosafety level-3 (BSL-3) laboratory, was completed in August 2017, and the facility was transferred to the Government of Kazakhstan on September 29, 2017. The CRL serves as the national diagnostic reference laboratory and an inter-ministerial agreement involving the Ministry of Health [National Scientific Center for Especially Dangerous Infections (NSCEDI)] with cooperation from the Ministry of Agriculture and the Ministry of Education and Science co-owns and operates the facility (2). The CRL operates BSL-3, animal biosafety level 3 (ABSL-3) and BSL-2 laboratories which have been designed to current international standards for biosafety and biosecurity.

DTRA BTRP's long standing engagement in the FSU region is also exemplified in Kazakhstan through cooperative biological research (CBR) under which 30 biosurveillance-related projects and studies have been implemented, utilizing over $25M in funding. In a previous paper, our team mapped these 30 projects, which spanned the periods 2005–2007, 2009–2014, and 2015–2018 (1). US contractors such as AECOM implemented these studies in concert with US and UK project collaborators and partner country scientists in Kazakhstan. More recently, DTRA BTRP has moved to a more traditional grant funding system where research collaborators submit proposals through a stand-alone competitive program. These opportunities are grouped into three categories by duration and approximate amount of funding (i.e., labor, material, and travel): 2–3 year, $1–3 M projects; 1–1.5 years, up to $1 M projects and 1 year, $100 K studies (only material and travel). In the absence of a current formal research office in Kazakhstan, through which an independent party typically manages the BTRP grant process, we recently and independently mapped research activities to illustrate the linkages, progression and evolution of the CBR program in Kazakhstan (1). From 2009 to 2014, three of the largest projects spawned eight follow-on projects, which emphasizes the interest from the partner country scientists in Kazakhstan in continuing previously funded cooperative research (1, 3). In addition, Kazakhstani scientists received training on topics such as biosafety, biosecurity, and laboratory diagnostics, which complemented their research activities. This integrated approach of parallel research and training serves as a model for all future cooperative activity.

**Figure 2:**
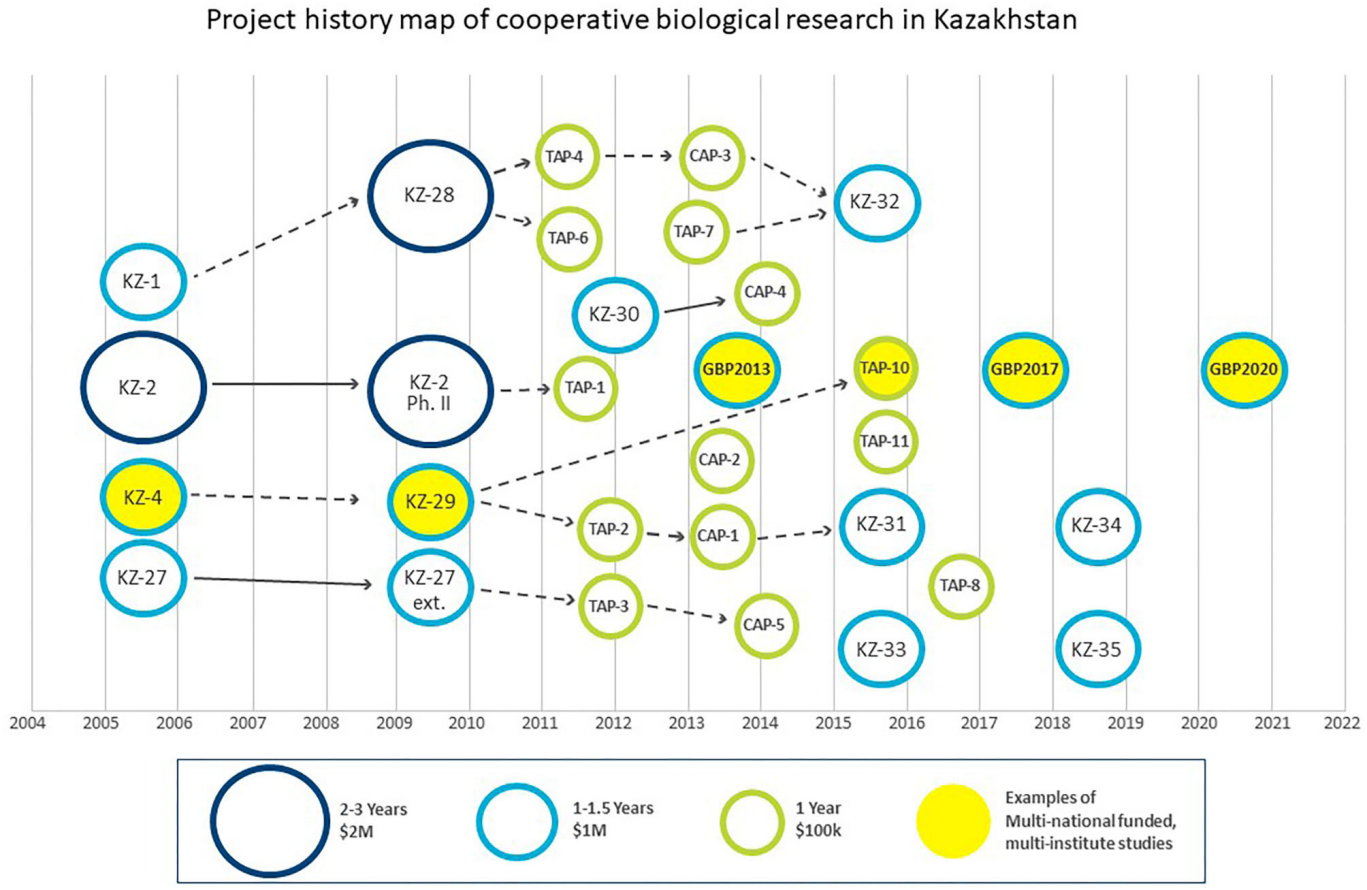
DTRA BTRP (KZ projects, TAP studies) and GBP have funded over 30 studies since 2005. UK IBSP directly funded UK specialists for KZ-4 and KZ-29. A number of research grants awarded in 2020 or later are not shown.

The **DTRA BTRP** and the **UK IBSP** have collaborated through agreements between the US Department of Defense and the UK Ministry of Defense. Similar to the situation with the DTRA BTRP, the UK Government funds its scientists on a “per project” basis, including technical assistance to partner country scientists in Kazakhstan through training and research capacity that UK specialists delivered. This agreement spawned two projects: KZ-4, a multi-viral pathogen study that ran from 2005 to 2007, that evolved into KZ-29, a broader tick-borne pathogen surveillance project targeting Crimean-Congo hemorrhagic fever (CCHF) virus, hantaviruses, tick-borne encephalitis (TBE) viruses and tick-borne rickettsiae that ran from 2009 to 2014 (1–3) (Figure 2). In 2020, DTRA awarded a new cooperative research grant for $1.5 M over three years to US, UK, and Kazakhstani scientists, who will continue study of the CCHF and TBE viruses endemic in Kazakhstan through novel sequencing and bioinformatics approaches. This activity illustrates the important continuity built in to the cooperative DTRA program, targeting issues of interest to all parties and involving many of the same personnel on all sides of the program.

Although **DTRA BTRP** and **GBP** have not formally collaborated, their respective efforts have developed very much in parallel (Figure 2). In 2013, the GBP was launched to foster responsible behavior in life sciences, strengthen national health security and focus on capacity development. From 2013 to 2021, the GBP supported three projects in Kazakhstan with a total expenditure of €2.5 M, utilizing the Bundeswehr Institute of Microbiology and the Deutsche Gesellschaft für Internationale Zusammenarbeit to implement this work through research and training. The initial effort was the establishment of a *German Kazakh Network for the diagnosis of infectious diseases caused by potential B-Agents* (2013–2016), followed by two projects called *German Kazakh Network for Biosafety and Biosecurity* (2017–2019 and 2020–2022) (4). Five national Kazakh institutions with expertise in arthropod and rodent vectors, especially dangerous pathogens or molecular biology were involved in the projects: the Kazakh National Medical University, the Scientific Practical Center for Sanitary and Epidemiological Expertise (SPCSEEM), NSCEDI including Taldykorgan and Uralsk Anti-plague Stations (UAPS), the Research Institute for Biological Safety Problems and the National Center for Biotechnology Almaty branch.

The **DTRA** and **GBP** projects have independently cooperated with three of the same institutes in Kazakhstan: SPCSEEM, NSCEDI, and UAPS. Examples of this cooperation include the aforementioned KZ-29 (as well as TAP-10, which was a 1-year study on tick-borne encephalitis virus, *Coxiella burnetii*, and *Brucella* species presence in livestock milk), in addition to the three GBP projects mentioned earlier (Figure 2). The KZ-29 project resulted in two publications (3, 5) and 21 conference presentations (1). Within the three German projects, two serological patient studies (Fevers of Unknown Origin), a tick-borne disease and a rodent-borne disease study were conducted. Scientific results and the progress of the projects were presented in 41 oral talks and 21 poster presentations at national and international scientific conferences. Work published from GBP involvement included articles describing the results of studies on CCHF and TBE viruses, rickettsiae and orthohantaviruses (6–9). As a result of awareness and post-project coordination of the DTRA and GBP programs on tick-borne diseases research (DTRA TAP-10, GBP), a joint publication on tick-borne encephalitis virus in North Kazakhstan is in final preparation.

Unquestionably, these three cooperative bio-research programs all resulted in better collaboration, increased communication, and significant investment in building scientific research capabilities in Kazakhstan (1, 3, 4). At the government-to-government level, there is agreement and interest to further this work which is demonstrated by the increase in the absolute number of projects. However, there have been no specific studies on what long-term impacts these programs have on the day-to-day approach to scientific research in Kazakhstan; thus it is hard to assess capability maturity and related performance and quality. Nevertheless, what our informal observations tell us is that increased awareness and visibility of these programs to the scientific community through conference presentations serve as good models for future similar international collaborations.

In view of the above uncertainties and to gain more formal insight into the impact of these international programs, our team developed a questionnaire that consisted of 21 questions, structured as yes or no, multiple choice and short answers.

The questions focused on an institute's research capability and were grouped in categories for demographics, standard processes for pursuing research funding and after-action and “lessons learned” processes. In addition, we asked about simple metrics, including the number of scientific conferences attended, the number of presentations given, and the number of publications per author per year, as well as the number of grants applied for in the last 5 years. The contact list was developed and the questionnaire was sent to 37 participants who were colleagues involved in bio-research and biosecurity programs as participants in at least one of the projects mentioned earlier. These individuals represented five institutes and two universities. For ease of completion, one of our team members who is a native Kazakh and Russian speaker translated the survey into Russian and this was provided as a Microsoft Word document attachment. Surveys were sent as requests to support this current manuscript construction and, in addition to scientists, recipients also included institute scientific secretaries who could best answer questions related to metrics. The University at Buffalo's Institutional Review Board reviewed the questionnaire which was submitted under IRB ID STUDY00004695, and deemed it to be “Exempt”from further IRB scrutiny.

The results represent a qualitative analysis from a limited sample (*n* = 11) of completed questionnaires (seven males and four females). The respondents represented five institutes and two universities that employed between 11 and 99 scientists in their home departments. The responses came from those at different positions of seniority, including institution director, laboratory head, and scientific secretary. Of the 11 respondents, 5 were in the 40–49 age category, 3 were in the 30–39 age category, 2 were in the 50–59 age category, and 1 was in the 60–69 age category.

When asked who decides which research funding opportunities are applied for, half responded that a laboratory/division/department head was the decision maker, followed by ministerial leadership and institute director. Respondents split on the question about whether their institute had an overall strategy for pursuing funding. The majority of respondents answered that their institute does not have a standard approach to identify and prioritize opportunities. The funding sources that the institutes pursued was consistent: mainly existing and historical sources (both domestic and foreign), followed by new opportunities, private, and foreign funding. Although the institutes represented employed up to 99 scientists, half responded that 11–49 of the scientists were involved in pursuing research funding and half responded that <10 scientists were involved.

Respondents stated that the three most important factors that the institutes considered when applying for research funding were the source, amount and scope. In addition, institutes also reported the importance of continuing existing research capabilities, ability to expand and applicability of the research to the institute's mission.

When applying for research funding, the majority of respondents stated that their institutes provided guidance, standard documents, tools, and training; however, two respondents stated the opposite. On the question of whether institutes held debriefs after a funding submission, all respondents stated this occurred internally but only half stated this occurred externally. Nearly all respondents stated that their institutes tracked applications and reviewed “lessons learned” to provide continuous improvement.

The majority of respondents stated they attended 1–3 international and 1–3 national conferences annually, as well as some local conferences. The number of publications varied from 2 to 18 distributed across different indices: Web of Science, Scopus, and the Russian Science Citation index. The number of proposals applied for in the past 5 years varied from 3 to 36 according to the respondent's job function.

## Discussion

As evidenced from funding records, US DTRA BTRP, GBP, and UK IBSP have each invested and engaged in cooperative research projects in Georgia and Kazakhstan. Kazakhstan's important role in biological threat reduction and biosecurity is reflected in the over $25 M in cooperative research projects awarded over the last 20 years. US DTRA BTRP milestones for infrastructure have been achieved through the construction and commissioning of two ZDLs from 2009 to 2010 and the Central Reference Laboratory in 2019; similar work was achieved in Tbilisi, Georgia (10). In discussions with our colleagues in Kazakhstan, the impact of these related programs is and will be substantial, particularly for Kazakhstan's current and next generation of scientists, confirmed through their more frequent participation at international conferences and a greater number of peer-reviewed publications in international journals. Our earlier compendium discussed a roadmap and framework to grow a research program and incorporate the value of simple metrics, such as number of publications, conference presentations, proposals submitted, and proposals secured (1) and this seems to be taking place. Successful partnerships with German, UK, and US researchers continue to raise awareness and visibility of this work in the global scientific community. Looking ahead, investments in these partnerships appear to be seen as relevant for the future of global biological threat reduction and public health. As mentioned, the DTRA BTRP program has started making awards through the mid-2020s for research proposals submitted through their annual funding opportunity calls.

There are many cultural, technical, and programmatic challenges associated with these cooperative bio-research programs that include the language barrier, lack of diagnostic capabilities and varying expectations among funders, recipients and stakeholders (1, 3, 4). Among the large funding programs, there is a challenge in managing the bureaucratic inertia of many implementing partners which often changes with different contract awards and can result in a lack of program continuity. Staff turnover on both sides leads to gaps in science and program knowledge. Regarding capability and capacity building, in earlier CBR projects short timelines and limited in-person interactions did not favor successful training and mentoring for developing *in vitro* diagnostic assays. Instead, commercial kits were purchased akin to an instant food option. While this was effective in the short term it is not sustainable in the long term. This is a “lesson learned” that has led to changes in newer contracts. Limitations in exchanging sample material with foreign partners, which is largely not permitted in Kazakhstan even for research (a measure of scientific transparency) were recently offset by permissions to exchange genomic sequencing data in an electronic form (4, 11). Most importantly, building capacity requires strong mentorship and trust among collaborators and partners who are working together on the same goals, objectives, and strategies.

Obtaining responses to our survey was challenging, especially since this was an unfunded effort that did not allow us to pay respondents. Our team addressed needs for access and ease-of-use by emailing a Word document with the survey in English and Russian, as noted earlier, and we assume that this was helpful. Although some respondents had participated in earlier surveys, we recognized that they did not generally have much experience with such requests for information. This may reflect the fact that, in many countries, grass roots opinions are rarely used in planning future research activities. Thus, the difficulty observed with participation in this survey may be the result of lack of experience especially from junior level scientists and continuing local practice. In reviewing our survey responses, we also observed that the nature of many of the answers was typical of institutions and organizations without standard or repeatable processes for capturing research funding.

Regarding peer reviewed publications, we learned that, in Kazakhstan, quality and prestige of a publication often depends on indices for Scopus, Web of Science, and the Russian Science Citation Index. One other encouraging aspect noted, however, is that more Kazakhstani partner country scientists are now first or senior authors in recent publications from cooperative studies. This is a welcome change, we believe brought about by the influence of cooperative international research programs, since in earlier years, it was US and UK project collaborators who initiated and authored joint publications. In recent years, Kazakh partner country scientists, especially those who have become proficient in English and actively collaborate in international programs, seem also to have become more successful in their scientific careers in general. Overall, this requires strong mentorship among collaborators to develop a goal and strategy. For example, if early work was fronted by scientists from the funder country, partner country scientists should be, and are being, consulted and invited to contribute as co-authors. A successful evolution of work, from the funders' perspective, would later show those partner country scientists as lead and senior authors with the earlier scientists from funder as middle authors. This transition is already evident, where strong teams can achieve these metrics through collaboration and, with limited resources, publish work not funded by foreign collaborators.

One final point worth mentioning was the lack of consensus among respondents on whether standard grant-getting procedures existed in their workplace, and who was responsible for developing and submitting proposals. Possessing such a structure and framework for grants is grounded within organizational values and objectives, such as striving to be the top research institution for a given region, country or sector. These high-level goals and pathways to maturing capabilities seem to be missing in many places in partner countries. If that can be changed, perhaps as a spin-off from cooperative research involvement, the route to developing better self-sustained research programs may in the future be accepted as having an agile playbook to capture external funding across multiple sources, as well as developing a diverse portfolio of capabilities and expert staff.

Recognizing and encouraging successful networks, such as the ones we have described, are important for growing and sustaining collaborations which in turn mature into sustainable new capabilities (11–14). In this work, singular projects among DTRA BTRP/IBSP and DTRA BTRP/GBP funded work created networks among common Kazakhstani partner scientists at common institutes. Ideally, diverse teams that represent not just various disciplines and backgrounds but also cross national boundaries help reinforce creativity and avoid one-dimensional group thought. The networks we have described also demonstrate strong communication among peers and a refreshing transparency, both of which promote ideas and trust, and work toward resolving difficult issues such as access to benefits and sharing challenges, to increase further scientific capability. In that context, while there is only limited collaborative activity among the foreign funding partners at the executive level, each partner's scientific staff who implement the research awards are in direct working contact with staff from other partners. Most importantly, however, is that foreign scientists have established long-term trusting relationships with Kazakh colleagues that continue outside of funding periods and have, in some cases, lasted for close to 20 years. Such relationships form the real core of cooperative research efforts, in that they have an enduring benefit, rather than a transient one, in which lines of communication remain open and where technical issues and future plans can be freely and honestly discussed.

## Future Prospects

In light of the COVID-19 pandemic, it is obvious that scientific and medical cooperation to enhance preparedness and response capability to global health events cannot be allowed to wait until a disaster is upon us. In the sense research starts with basic efforts that lead to applied and translational activity, and infectious disease surveillance provides the data that drive the process. These efforts all require investment in training and research, such as we have described for the Cooperative Biological Threat Reduction initiatives in this article.

There are numerous other cooperative research programs such as NIH Fogarty and ICAP that should be continued to reinforce existing networks and keep generate awareness for such activities. In Kazakhstan, there are additional instances of international collaboration other than the three programs we have described. For example, a memorandum of understanding was signed in 2019 between Ohio State University and the Kazakh National Agrarian University to collaborate on COVID-19 vaccine work, while the International Science and Technology Committee (ISTC) has been active there for many years, approving research work funded from its multinational contributors. Kazakhstani scientists also mentioned positive experiences through two international exchanges programs. The US Borlaug Fellowship Program offers a collaborative research program by pairing early-career scientists with a US mentor to study agricultural and veterinary topics. The Bolashak International Scholarship funded by the Government of Kazakhstan provides all-expenses-paid studies at the world's top universities and recipients return to Kazakhstan to provide 5 years of work service. The Ministry of Education and Science of Kazakhstan also has a Strategic Plan for 2020–2024 specific to further developing science under three priorities with various measures similar to what we have described (15). The benefits of these experiences help develop the next generation of leaders who can further mature their respective research programs in Kazakhstan, based on the formation of cooperative scientific arrangements.

## Author Contributions

KY, KT, FP, and JH developed the concept. EM, YS, ZS, AS, SF, SE, RH, and AR contributed to the manuscript. All authors reviewed and confirmed this work.

## Conflict of Interest

FP was employed by the company EpiPointe, LLC. The remaining authors declare that the research was conducted in the absence of any commercial or financial relationships that could be construed as a potential conflict of interest.

## Publisher's Note

All claims expressed in this article are solely those of the authors and do not necessarily represent those of their affiliated organizations, or those of the publisher, the editors and the reviewers. Any product that may be evaluated in this article, or claim that may be made by its manufacturer, is not guaranteed or endorsed by the publisher.
